# Psychometric properties of an Arabic translation of the brief version of the difficulty in emotion regulation scale (DERS-16)

**DOI:** 10.1186/s40359-023-01117-2

**Published:** 2023-03-15

**Authors:** Feten Fekih-Romdhane, Gaelle Kanj, Sahar Obeid, Souheil Hallit

**Affiliations:** 1 The Tunisian Center of Early Intervention in Psychosis, Department of Psychiatry “Ibn Omrane”, Razi Hospital, Manouba, 2010 Tunisia; 2 Faculty of Medicine of Tunis, Tunis El Manar University, Tunis, Tunisia; 3 School of Arts and Sciences, Holy Spirit University of Kaslik, Jounieh, P.O. Box 446, Lebanon; 4 Social and Education Sciences Department, School of Arts and Sciences, Lebanese American University, Jbeil, Lebanon; 5 School of Medicine and Medical Sciences, Holy Spirit University of Kaslik, Jounieh, P.O. Box 446, Lebanon; 6 Psychology Department, College of Humanities, Efat University, Jeddah, 21478 Saudi Arabia; 7 Applied Science Research Center, Applied Science Private University, Amman, Jordan; 8 Research Department, Psychiatric Hospital of the Cross, Jal Eddib, Lebanon

**Keywords:** DERS-16, Short version, Emotion regulation, Arabic, Psychometric properties

## Abstract

**Background:**

The current study aimed to examine the psychometric properties of an Arabic translation of the short form of the Difficulty in Emotion Regulation Scale (DERS-16) in an Arabic-speaking population-based adult sample in Lebanon. In particular, the factorial structure, composite reliability, convergent validity and gender invariance were investigated.

**Methods:**

A total of 411 Lebanese adult participants (mean age of 32.86 ± 11.98 years, 75.4% females) took part of this cross-sectional web-based study. The forward and backward translation method was applied.

**Results:**

Findings revealed good internal consistency of the Arabic DERS-16 total scale and five subscales (McDonald’s ω ranging from 0.81 to 0.95). Confirmatory factor analyses confirmed the five-factor solution of the scale and demonstrated strong measurement invariance across gender at the configural, metric, and scalar levels. No significant differences were found in all DERS-16 domains between men and women participants. Finally, the DERS-16 scores and sub-scores showed strong correlations with the Borderline Personality Questionnaire (r > .40), thus indicating its convergent validity.

**Conclusion:**

Overall, the present findings suggest that the Arabic version of the DERS-16 may be a reliable and valid self-report measure that assesses ER difficulties as a multidimensional construct. Making the Arabic version of the DERS-16 available will hopefully strengthen its utilization for clinical and research purposes to benefit the millions of Arabic-speaking people worldwide.

## Introduction

Emotion regulation (ER) is a multidimensional concept that can be defined as one’s ability to recognize, assess, respond to, and monitor the expression of their felt emotions, in order to reach their own goals [1–3]. Due to its major clinical relevance, ER has attracted a growing research interest during the last years [4, 5], which was designated to as the “affect revolution” [6]. Indeed, ER provide valuable information on the ways and behaviors that can improve or maintain situational adaptability [7], and lead to greater functioning and psychological wellbeing [5, 8–10]. When deficient, however, ER has consistently been demonstrated to relate to a wide range of negative behavioral and psychological outcomes [9, 11–14], including depression and anxiety disorders [15, 16], posttraumatic stress disorder [17], eating disorders [18], substance use [19], and borderline personality disorder [20–23]. Evidence has also supported the role of ER in the treatment of multiple mental health problems [24–26]. These substantial implications motivated the development of numerous measures to assess the ER construct and its different facets (e.g., [27–32]). Nevertheless, a unified and standardized way to evaluate multi-domain aspects of ER across populations is still lacking and strongly needed [13, 33].

One comprehensive, theoretically-supported, and widely used measure of ER difficulties is the Difficulties in Emotion Regulation Scale (DERS; [34]). The DERS is a 36-item self-report scale that has been originally developed by Gratz and Roemer in 2004 among US undergraduate students; and contains six factors, i.e., lack of emotional clarity, difficulties in goal directed behavior, impulse control difficulties under distress, limited access to emotion regulation strategies, and non-acceptance of emotional responses [34]. The scale has then proven to be useful and reliable for clinical and treatment applications [12, 35, 36]. This has therefore encouraged the development of three shorter forms for easier and more convenient use, i.e., a 18-item (DERS-18) [37], a 17-item (DERS-SF) [38] and a 16-item (DERS-16 ) [39] versions. Among these versions, the briefest one (i.e. DERS-16) exhibited the highest item total correlations [40]. In addition, the DERS-16 showed excellent psychometric properties both in clinical and non-clinical samples [39]. In particular, the scale yielded excellent internal consistency (Cronbach’s α = 0.92), good test-retest reliability (ρI = 0.85; p < .001), appropriate construct validity equivalence (as evidenced through correlations with the original 36-item DERS), as well as adequate discriminant validity (as attested by significant correlations of DERS-16 scores with measures of negative emotionality, mindfulness, experiential avoidance, psychiatric symptoms, deliberate self-harm, borderline symptoms, and alcohol use disorder) [39]. When designing the DERS-16, Bjureberg et al. decided to exclude the Awareness factor due to its weak psychometric qualities [36, 41–43], resulting thus in a five-factor structure model [39]. Since its development, several studies sought to investigate the DERS-16’s psychometric characteristics across different settings and in different populations, such as adolescents with non-suicidal self-injury [44, 45], adolescents and adults with severe mental illnesses [46], community adults [47], adults with substance use disorders [48, 49] and emotional disorders [36]. The DERS-16 has also been cross-validated in several languages and cultures, including Finnish [50], Brazilian [43], Turkish [51], Swedish [39], and Persian [52]. However, there is currently no version of the DERS adapted for the Arabic-speaking population.

The present study focused on examining the appropriateness of the Arabic version of the DERS-16 in a non-clinical Lebanese population, for several reasons. First, there had been very limited research on ER in Arab contexts [53], likely due to a lack of cross-culturally adapted and valid measures. The Emotion Regulation Questionnaire (ERQ; [27]) is the only ER measure available in Arabic to date. It has been previously validated in Lebanese community adults [53, 54], Saudi Children and Adolescents [55], and Iraqi nursing students [56]. While DERS enables to capture broad ER domains, the ERQ is a narrower measure specifically focused on cognitive strategies; consequently, DERS demonstrated more usefulness in clinical settings compared to ERQ [47]. Second, the DERS-16 is highly advantageous because of its brevity. This is of major importance, particularly in the low- and middle-income Arab countries, as it offers reduced administration time, respondents’ burden and costs. This has led several authors to recommend its selection for use over all DERS versions (i.e. the full-length and three short forms) when aiming at assessing difficulties in ER (e.g., [40]). Third, all versions of the DERS have been developed in Western contexts and English-speaking populations. However, research pointed to the culturally-dependent nature of ER [57] and the significant differences between western and Arab samples regarding how ER strategies are used and how they relate to psychopathology [58], [59]. It remains thus unknown whether the DERS can be appropriate and useful in people from Arab cultural backgrounds and settings. For all these reasons, it becomes obvious there is a strong need for adequately translated and validated version of the DERS-16 into the Arabic language.

To this end, the current study aimed to examine the psychometric properties of an Arabic translation of the DERS-16 in an Arabic-speaking population-based adult sample in Lebanon. In particular, the factorial structure, composite reliability, convergent validity and gender invariance were investigated. We hypothesized that the Arabic DERS-16 will (1) show high internal consistency and convergent validity, (2) replicate the five-factor structure of the initial version, and (3) demonstrate measurement invariance across gender.

## Methods

### Procedures

All data were collected via a Google Form link, between May and July 2022. The project was advertised on social media and included an estimated duration. Inclusion criteria for participation included being a resident and citizen of Lebanon of adult age (≥ 18 years). Excluded were those who refused to fill out the questionnaire. Internet protocol (IP) addresses were examined to ensure that no participant took the survey more than once. After providing digital informed consent, participants were asked to complete the instruments described above, which were presented in a pre-randomised order to control for order effects. The survey was anonymous and participants completed the survey voluntarily and without remuneration.

### Translation Procedure

A common procedure of back-translation was followed in the present study, in which a text is translated from a source into a target language, and then independently back-translated into the source language by a second interpreter. Therefore, the English version of the DERS was translated to Arabic by a Lebanese translator who was completely unrelated to the study. Afterwards, a Lebanese psychologist with a full working proficiency in English, translated the Arabic version back to English. To evaluate the accuracy of the translation, the initial and back-translated English versions were compared [60, 61]; and any inconsistencies were detected and eliminated by a committee composed of the research team and the two translators. The translated Arabic version was pretested in a sample of 20 persons from the target population to ensure understanding of the questions [62].

### Measures

#### Difficulty in Emotion Regulation Scale (DERS-16).

It is a 16-item scale that assesses difficulties in emotion regulation [39]. Items are graded using a 5-point Likert scale. Higher scores reflect more emotion regulation difficulties. Within the scale are five subscales: lack of emotional clarity (e.g., “I am confused about how I feel”; 2 items), inability to engage in goal-directed behaviours when distressed (e.g., “When I am upset, I have difficulty getting work done”; 3 items), difficulties controlling impulsive behaviours when distressed (e.g., “When I am upset, I become out of control”; 3 items), non-acceptance of negative emotions (e.g., “When I am upset, I feel like I am weak”; 3 items), and limited access to emotion regulation strategies perceived as effective (e.g., “When I am upset, my emotions feel overwhelming”; 5 items).

#### Borderline Personality Questionnaire.

The BPQ was developed by Poreh et al. [63] and is a self-report scale composed of 80 items, with a true/false option. BPQ Borderline personality traits tested for reliability and validity are evaluated according to DSM-IV criterions. BPQ scale has nine subscales which are Impulsivity, Instability in affect, Abandonment, Relationships, Self-Image, Suicide/Self-Mutilation Behavior, Emptiness, intense Anger, and Psychosis-like Cases. Higher scores indicate the presence of a borderline personality. The Arabic version of the BPQ has been used [64]. The BPQ total scores yielded a McDonald’s ω of 0.94 in the present sample.

#### Demographics.

Participants were asked to provide their demographic details consisting of age, sex, highest educational attainment, region of living, marital status and the Household Crowding Index (HCI); the latter reflecting the socioeconomic status of the family [65], is the ratio of the number of persons living in the house over the number of rooms in it (excluding the kitchen and the bathrooms).

### Analytic strategy

#### Confirmatory factor analysis.

There were no missing responses in the dataset. We used data from the total sample to conduct a CFA using the SPSS AMOS v.26 software. The minimum sample size to conduct a confirmatory factor analysis ranges from 3 to 20 times the number of the scale’s variables [66]. Therefore, we assumed a minimum sample of 320 participants needed to have enough statistical power based on a ratio of 20 participants per one item of the scale, which was exceeded in our sample. Our intention was to test the original model of the DERS-16 scores (i.e., five-factor model). Parameter estimates were obtained using the maximum likelihood method and fit indices. Additionally, evidence of convergent validity was assessed in this subsample using the Fornell-Larcker criterion, with average variance extracted (AVE) values of ≥ 0.50 considered adequate [67] and meaning that a latent variable is able to explain more than half of the variance of its indicators on average (i.e., items converge into a uniform construct). To carry out a CFA, the following assumptions must be met: 1) inter-item correlation (the average correlation should be between 0.20 and 0.40), item-to-factors correlation (coefficient > 0.4, suggests convergent validity of items within the same factors) and inter-factors correlation (coefficient of > 0.4 support convergent validity).

#### Gender invariance.

To examine gender invariance of the DERS-16 scores, we conducted multi-group CFA [68] using the total sample. Measurement invariance was assessed at the configural, metric, and scalar levels [69]. Configural invariance implies that the latent scales variable(s) and the pattern of loadings of the latent variable(s) on indicators are similar across gender (i.e., the unconstrained latent model should fit the data well in both groups). Metric invariance implies that the magnitude of the loadings is similar across gender; this is tested by comparing two nested models consisting of a baseline model and an invariance model. Lastly, scalar invariance implies that both the item loadings and item intercepts are similar across gender and is examined using the same nested-model comparison strategy as with metric invariance [68]. Following the recommendations of Cheung and Rensvold (2002) [70] and Chen (2007) [68], we accepted ΔCFI ≤ 0.010 and ΔRMSEA ≤ 0.015 or ΔSRMR ≤ 0.010 (0.030 for factorial invariance) as evidence of invariance.

#### Further analyses.

Composite reliability in both subsamples was assessed using McDonald’s (1970) ω, with values greater than 0.70 reflecting adequate composite reliability [71]. McDonald’s ω was selected as a measure of composite reliability because of known problems with the use of Cronbach’s α (e.g., [72]). To assess convergent and criterion-related validity, we examined bivariate correlations between DERS and BPQ scores. Based on Cohen (1992) [73], values ≤ 0.10 were considered weak, ~ 0.30 were considered moderate, and ~ 0.50 were considered strong correlations.

## Results

### Sociodemographic and other characteristics of the sample

Four hundred eleven participants were included in this study, with a mean age of 32.86 ± 11.98 years and 75.4% females. Based on a cut-off point of 1.5 SD, the results showed that 35 (8.5%) participants had underdiagnosed borderline personality disorder. Other descriptive statistics of the sample can be found in Table 1.

**Table 1:** Sociodemographic and other characteristics of the sample (N = 411)

Variable	N (%)
Sex	
Male	101 (24.6%)
Female	310 (75.4%)
Marital status	
Single	254 (61.8%)
Married	157 (38.2%)
Education level	
Secondary or less	39 (9.5%)
University	372 (90.5%)
Governorate	
Beirut	40 (9.7%)
Mount Lebanon	295 (71.8%)
North	26 (6.3%)
South	27 (6.6%)
Bekaa	23 (5.6%)
	**Mean ± SD**
Age (years)	32.86 ± 11.98
Household crowding index (persons/room)	0.93 ± 0.44
BPQ score	24.78 ± 14.22
DERS score	34.00 ± 13.02

### Inter-item, item-factors and inter-factors correlations

The description of the Inter-item, item-factors and inter-factors correlations are summarized in Tables 2, 3 and 4. Assumptions were met except for the inter-item correlations that showed an average > 0.4, revealing good convergent validity of the scale.

**Table 2:** Inter-item correlations

	1	2	3	4	5	6	7	8	9	10	11	12	13	14	15	
1. DERS 1	1															
2. DERS 2	0.77***	1														
3. DERS 3	0.49***	0.48***	1													
4. DERS 4	0.40***	0.44***	0.51***	1												
5. DERS 5	0.55***	0.54***	0.47***	0.51***	1											
6. DERS 6	0.56***	59***	0.50***	0.50***	0.75***	1										
7. DERS 7	0.51***	0.50***	0.74***	0.48***	0.53***	0.57***	1									
8. DERS 8	0.49***	0.50***	0.55***	0.72***	0.60***	0.65***	0.63***	1								
9. DERS 9	0.43***	0.40***	0.28***	0.31***	0.48***	0.45***	0.35***	0.41***	1							
10. DERS 10	0.55***	0.54***	0.47***	0.43***	0.57***	0.56***	0.56***	0.55***	0.63***	1						
11. DERS 11	0.44***	0.45***	0.42***	0.64***	0.51***	0.51***	0.47***	0.67***	0.44***	0.52***	1					
12. DERS 12	0.50***	0.50***	0.49***	0.46***	0.59***	0.59***	0.57***	0.58***	0.46***	0.58***	0.57***	1				
13. DERS 13	0.46***	0.48***	0.36***	0.39***	0.46***	0.52***	0.46***	0.43***	0.53***	0.60***	0.44***	0.50***	1			
14. DERS 14	0.53***	0.53***	0.45***	0.39***	0.59***	0.63***	0.52***	0.54***	0.59***	0.67***	0.53***	0.59***	0.73***	1		
15. DERS 15	0.48***	0.49***	0.62***	0.49***	0.53***	0.59***	0.71***	0.60***	0.42***	0.60***	0.51***	0.58***	0.47***	0.57***	1	
16. DERS 16	0.51***	0.52***	0.56***	0.46***	0.59***	0.65***	0.59***	0.57***	0.44***	0.55***	0.51***	0.56***	0.49***	0.64***	0.67***	

***p < .001

**Table 3:** Inter-factor correlations

	1	2	3	4	5
1. DERS clarity	1				
2. DERS goals	0.59***	1			
3. DERS impulse	0.54***	0.66***	1		
4. DERS strategies	0.68***	0.73***	0.71***	1	
5. DERS non-acceptance	0.60***	0.58***	0.58***	0.76***	1

***p < .001

**Table 4:** Item-factor correlations

	DERS clarity	DERS goals	DERS impulse	DERS strategies	DERS non-acceptance
1. DERS 1	0.94***				
2. DERS 2	0.94***				
3. DERS 3		0.89***			
4. DERS 4			0.89***		
5. DERS 5				0.84***	
6. DERS 6				0.87***	
7. DERS 7		0.91***			
8. DERS 8			0.90***		
9. DERS 9					0.84***
10. DERS 10					0.88***
11. DERS 11			0.86***		
12. DERS 12				0.80***	
13. DERS 13					0.83***
14. DERS 14				0.83***	
15. DERS 15		0.87***			
16. DERS 16				0.83***	

***p < .001

### Confirmatory factor analysis of the DERS scale

CFA indicated that fit of the three-factor model of the DERS-16 was acceptable: χ^2^/df = 397.22/94 = 4.22, RMSEA = 0.088 (90% CI 0.079; 0.097), SRMR = 0.044, CFI = 0.935, TLI = 0.917. When adding a correlation between residuals of items 13 and 14, the fit indices improved as follows: χ2/df = 321.20/93 = 3.45, RMSEA = 0.077 (90% CI 0.068, 0.086), SRMR = 0.040, CFI = 0.951, TLI = 0.937. The standardised estimates of factor loadings were all adequate (see Table 5). The convergent validity for this model was borderline, as AVE = 0.675.

**Table 5:** *Items of the difficulty in emotion regulation scale (DERS-16) in English and Factor Loadings Derived from the Confirmatory Factor Analysis (CFA) in the total sample*

	Total
Factor 1	
1	0.89
2	0.89
Factor 2	
3	0.70
7	0.80
15	0.83
Factor 3	
4	0.83
8	0.88
11	0.86
Factor 4	
5	0.76
6	0.83
12	0.76
14	0.79
16	0.72
Factor 5	
9	0.81
10	0.82
13	0.83

### Composite reliability

Composite reliability of scores was adequate in the total sample for the total scale (ω = 0.95), clarity (ω = 0.87), goals (ω = 0.87), impulse (ω = 0.86), strategies (ω = 0.89), and non-acceptance (ω = 0.81).

### Gender invariance

As reported in Table 6, all indices suggested that configural, metric, and scalar invariance was supported across gender. Given these results, we computed an independent-samples *t*-test to examine gender differences in terms of DERS-16 total scale and subscales scores; no significant difference was found between men and women in terms of all DERS-16 scores (Table 7).

**Table 6:** Measurement Invariance of the difficulty in emotion regulation scale (DERS-16) Across Gender in the total sample

Model	χ²	*df*	CFI	RMSEA	SRMR	Model Comparison	Δχ²	ΔCFI	ΔRMSEA	ΔSRMR	Δ*df*	*p*
Configural	432.11	186	0.948	0.057	0.056							
Metric	438.25	197	0.949	0.054	0.056	Configural vs. metric	6.14	0.001	0.003	< 0.001	11	0.863
Scalar	453.84	208	0.948	0.053	0.056	Metric vs. scalar	15.59	0.001	0.001	< 0.001	11	0.157

*Note.* CFI = Comparative fit index; RMSEA = Steiger-Lind root mean square error of approximation; SRMR = Standardised root mean square residual.

**Table 7:** Comparison between sexes in terms of the difficulty in emotion regulation scale (DERS-16) total scale and subscales scores in the total sample

	DERS total score	Clarity	Goals	Impulse	Strategies	Non-acceptance
Males	34.82 ± 13.45	4.27 ± 1.98	7.68 ± 2.90	6.19 ± 2.98	10.26 ± 4.74	6.41 ± 2.96
Females	34.16 ± 13.46	4.00 ± 1.89	7.48 ± 3.08	6.06 ± 2.86	10.36 ± 4.89	6.26 ± 2.95
t	0.427	1.251	0.571	0.406	0.179	0.444
*p*	0.670	0.212	0.568	0.685	0.858	0.657

Numbers in bold indicate significant p-values.

### Convergent and criterion-related validity

To assess the validity of the scores, we examined bivariate correlations between all DERS-16 scores and borderline personality using the total sample. All DERS-16 scores were significantly associated with more borderline personality. Older age was significantly associated with lower DERS-16 scores (Table 8).

**Table 8:** Correlations of the difficulty in emotion regulation scale (DERS-16) total scale and subscales scores with the other measures in the total sample

	1	2	3	4	5	6	7	8	9
1. DERS-16 total score	1								
2. Clarity	0.77***	1							
3. Goals	0.84***	0.59***	1						
4. Impulse	0.82***	0.54***	0.66***	1					
5. Strategies	0.94***	0.68***	0.73***	0.71***	1				
6. Non-acceptance	0.83***	0.60***	0.58***	0.58***	0.76***	1			
7. BPQ total score	0.63***	0.54***	0.50***	0.54***	0.63***	0.44***	1		
8. Age	− 0.35***	− 0.37***	− 0.33***	− 0.22***	− 0.30***	− 0.29***	− 0.24***	1	
9. Household crowding index	0.03	0.05	0.02	0.05	− 0.003	0.05	− 0.01	− 0.10*	1

*p < .05; **p < .01; ***p < .001. BPQ: Borderline Personality Questionnaire.

## Discussion

This is the first study to describe the psychometric properties of the DERS-16 in an Arabic-speaking population. As expected, the Arabic DERS-16 demonstrated good internal consistency and convergent validity. The results also showed that the five-factor model produced a good fit and was invariant across gender. Overall, the present findings suggest that the Arabic version of the DERS-16 may be a reliable and valid self-report measure that assesses ER difficulties as a multidimensional construct. On account of its brevity and easier administration, the DERS-16 may be particularly suitable for use in Arab settings that are afflicted by financial and resource scarcity along with a shortage of psychometrically-sound research tools [74].

Our investigation of the psychometric properties revealed good internal consistency of the Arabic DERS-16 total scale and five subscales (McDonald’s ω ranging from 0.81 to 0.95), which is in agreement with the original validation study [39] as well as other previous studies in different settings and contexts. Most of these studies, however, used Cronbach’s alphas coefficients to examine internal consistency of the DERS-16. For instance, Sörman et al. found high reliability of the English version of the DERS-16 scale among US community adults (Cronbach’s Alpha coefficients ranging from 0.71 to 0.92) [47]. Similar properties have been reported among non-clinical adults using other linguistic versions (i.e. Brazilian, alphas > 0.80 [43]; Turkish, alphas > 0.78 [51]; Finnish, alphas > 0.70 [50]), and in clinical samples (e.g., US adult outpatients with depression, anxiety and stress-related disorders [36]). One strength of our study over previous studies is the use of McDonald’s ω, which has proven more advantageous in estimating the internal consistency of multidimensional research tools compared to Cronbach’s alpha [75].

The present findings from the CFA confirmed the five-factor solution of the DERS-16, thus providing additional support to the cross-cultural validity of the scale. The five-factor structure of the DERS-16 has been consistently supported in the original scale development study [39], as well as in Finnish [50] and Brazilian [43] population-based samples. Likewise, other studies have demonstrated that the DERS-16 displayed a five-factor structure in Turkish [51], Persian [52], and Australian [40] undergraduate students. Beyond its factorial structure, the Arabic DERS-16 also demonstrated strong measurement invariance across gender at the configural, metric, and scalar levels. This implies that the multidimensional structure of the scale is equally applicable in Arabic-speaking male and female samples. The original validation study could not provide information on the appropriateness for use with both genders due to involving a majority of female participants [39]. We could find only one previous study that has documented similar evidence for invariance by gender groups on the basis of the subscales of the DERS-16 [51]. Furthermore, we could find no significant differences in all DERS-16 domains between men and women participants. While our results were consistent with few previous studies (e.g., [76, 77]), the existing research on gender-related differences in ER have generally revealed mixed findings. Some studies found that women tend to exhibit more difficulties in some ER dimensions (especially in the Goals and Non-acceptance dimensions) [43, 50, 51, 78, 79], whereas others found that these differences were no longer significant when considering the total DERS scores [17, 34, 80]. Gender differences regarding ER strategies are shaped by cultural norms and societal gender roles [81], which may explain these inconsistencies. Further studies exploring measurement invariance and gender differences in the various versions available of the DERS-16 in different cultural backgrounds are needed to clarify these discrepancies.

The DERS-16 scores and sub-scores showed strong correlations with the Borderline Personality Questionnaire, thus indicating its convergent validity. As noted earlier, there is sufficient evidence that DERS scores are linked to many forms of psychopathology [82–85], including personality disorder [20]. In addition, ER difficulties have been shown to overlap with a range of behavioral problems that characterize people with borderline personality traits, such as substance use [19, 39, 86], deliberate self-harm [87], and risky sexual behavior [86, 88]. Overall, the high level of convergence between the BPQ and the DERS-16 demonstrate that the latter allows identifying aspects of ER difficulties that are relevant to borderline personality traits. These findings provide further support to the clinical utility of the DERS-16. These findings provide further support to the clinical utility of the DERS-16, and strengthens the evidence of its adequacy in clinical populations as a screening and monitoring measure [47].

### Study limitations and research perspectives

This study has certain strengths that deserve to be highlighted, including the use of McDonald’s ω to examine internal consistency and the evaluation of gender invariance. We also extend previous research on the cross-cultural validity of the 16-item five-structure version of the DERS, by providing its Arabic validated translation. At the same time, our study has a number of limitations that need to be discussed. First, we only included community participants, thus limiting the generalization of our findings. Additional studies need to confirm the appropriateness of the use of the Arabic DERS-16 in clinical samples. Second, we used a cross-sectional design, which does not enable causal inference. Third, some psychometric properties of the Arabic DERS-16 (such as test-retest reliability and divergent validity) have not been assessed in the context of the present study, and need to be subject of future research. No cognitive criteria were taken into consideration while enrolling participants. Finally, Prior cross-cultural research has shown that ER strategies are present but used differently across various Arab countries and contexts [89], highlighting the need to further validate the DERS-16 in Arabic-speaking populations other than the Lebanese, to confirm its cross-cultural robustness.

## Conclusion

The present findings have shown that the original five-factor structure of the DERS-16 based on English-speaking Western samples remains stable in our Lebanese Arabic-speaking sample and yielded strong factorial invariance across gender groups. In addition, results demonstrated the high validity and reliability of the scale, overall preliminarily suggesting that it is a promising measure for capturing clinically relevant ER difficulties in Arab settings. Making the Arabic version of the DERS-16 available will hopefully strengthen its utilization for clinical and research purposes to benefit the millions of Arabic-speaking people worldwide.

## Data Availability

All data generated or analyzed during this study are not publicly available due the restrictions by the ethics committee (data are owned by the Psychiatric Hospital of the Cross). The dataset supporting the conclusions is available upon request to Ms. Rana Nader (rnader@naderlawoffice.com), a member of the ethics committee at the Psychiatric Hospital of the Cross.
